# Contributions of Immune Cells and Stromal Cells to the Pathogenesis of Systemic Sclerosis: Recent Insights

**DOI:** 10.3389/fphar.2022.826839

**Published:** 2022-02-03

**Authors:** Bingying Dai, Liqing Ding, Lijuan Zhao, Honglin Zhu, Hui Luo

**Affiliations:** ^1^ Department of Rheumatology and Immunology, Xiangya Hospital, Central South University, Changsha, China; ^2^ Provincial Clinical Research Center for Rheumatic and Immunologic Diseases, Xiangya Hospital, Changsha, China; ^3^ National Clinical Research Center for Geriatric Disorders, Xiangya Hospital, Changsha, China

**Keywords:** systemic sclerosis, ScRNA-seq, immune cells, fibroblasts, endothelial cells

## Abstract

Systemic sclerosis (SSc) is a multisystem rheumatic disease characterized by vascular dysfunction, autoimmune abnormalities, and progressive organ fibrosis. A series of studies in SSc patients and fibrotic models suggest that immune cells, fibroblasts, and endothelial cells participate in inflammation and aberrant tissue repair. Furthermore, the growing number of studies on single-cell RNA sequencing (scRNA-seq) technology in SSc elaborate on the transcriptomics and heterogeneities of these cell subsets significantly. In this review, we summarize the current knowledge regarding immune cells and stromal cells in SSc patients and discuss their potential roles in SSc pathogenesis, focusing on recent advances in the new subtypes by scRNA-seq.

## 1 Introduction

Systemic sclerosis (SSc) is an autoimmune connective tissue disease that can be mainly divided into limited cutaneous SSc (lcSSc) and diffuse cutaneous SSc (dcSSc) according to the extent of skin involvement (LeRoy et al., 1988). In patients, lcSSc mainly manifests as a thickening and hardening of the distal acral skin, often related to positive anti-centromere antibodies. Whereas dcSSc in patients also involves the proximal limbs and trunk which are prone to develop visceral organ complications, commonly associated with positive anti-topoisomerase I (anti-Scl-70) or anti-RNA polymerase III antibodies (Steen 2005).

SSc has three major pathological characteristics, including vasculopathy, immune dysregulation, and connective tissue fibrosis. Dysfunction of endothelial cells (ECs) is a key event leading to SSc microangiopathy (Mostmans et al., 2017). Activated ECs are able to recruit inflammatory cells by secreting adhesion molecules and E-selectin or stimulate extracellular matrix (ECM) production by connective tissue growth factor (CTGF) or other pro-fibrotic mediators. Innate and adaptive immune responses play a prominent part in the progression of SSc (Lech and Anders 2013; Allanore et al., 2015). Substantial evidence uncovers the link between the immune system and fibrosis. For instance, specific serum autoantibodies and pro-fibrotic cytokines like IFN-*α*, IL-4, and IL-13 and the transforming growth factor-beta (TGF-β) secreted by inflammatory cells, as well as abnormal inflammatory signatures [such as type-I interferon (IFN) signature] appear in the blood or target organs of SSc patients (Allanore et al., 2015). In addition, *in situ* hybridization has found that fibroblasts adjacent to the infiltrated inflammatory cells are more likely to synthesize collagen, implying the recruitment to fibroblasts (Fleischmajer et al., 1977; Kähäri et al., 1988). In SSc, alteration of activation, proliferation, and differentiation in the fibroblast occupy kernel status in tissue fibrosis. These cells release different kinds of chemokines that promote or inhibit the recruitment of circulating immune cells. Moreover, the activation of fibroblasts prevents T-cell apoptosis and enhances the sustainability of T-cell function (Pilling et al., 1999; O'Reilly et al., 2012). Interactions between the fibroblasts and immune cells often lead to the amplification of ECM production. In addition, abundant works have emphasized the process on injured ECs transdifferentiating toward myofibroblasts known as endothelial-to-mesenchymal transition (EndMT), which is also responsible for the expansion of the fibroblast pool (Xu et al., 2011; Jimenez 2013; Ebmeier and Horsley 2015).

In recent years, droplet-based single-cell RNA sequencing (scRNA-seq) has been developed at a rapid pace. In SSc, scRNA-seq reveals novel or rare cell types with great precision and characterizes their developmental trajectory. Profiling cell landscapes by scRNA-seq depicts the entire cell compositions under the conditions of physiology and pathology. Moreover, scRNA-seq allows researchers to discern the potential roles of differentially expressed genes in individual cells and their involvement in signaling pathways which sheds light on the pathogenic mechanism of SSc. There are currently plentiful studies on scRNA-seq targeting monocytes, macrophages, dendritic cells (DCs), T cells, fibroblasts, and ECs in SSc.

In this review, we primarily focus on the recent insights into the characteristics and functions of immune cells and stromal cells in various tissues including the blood, lungs, and skin in SSc patients by scRNA-seq and traditional methods. Therefore, it will greatly benefit to facilitate our comprehension of pathogenic mechanisms and the development of subsequent treatments.

## 2 Innate Immune Cells

The innate immune system has been shown to be activated at various stages of SSc. Generally, pattern recognition receptors, such as Toll-like receptors (TLRs) in SSc, identify damage-associated molecular patterns and pathogen-associated molecular patterns, which trigger an inflammatory response (Martin 2014) and could lead to a fibrotic state ultimately (Dowson et al., 2017). Myeloid cells, such as monocytes, macrophages, and DCs, are representative of innate immunity and play a significant role in the onset and progression of SSc (Satoh et al., 2017; Ah Kioon et al., 2018; Bhandari et al., 2020). Monocytes participate in fibrosis progression by affecting inflammatory responses and differentiating into macrophage or fibroblast-like cells (Shi and Pamer 2011). The human monocytes can be divided into three subtypes according to the expression of membrane molecules: classical monocytes (CD14^++^CD16^−^), nonclassical monocytes (CD14^+^CD16^++^), and intermediate monocytes (CD14^+^CD16^+^) (Passlick et al., 1989; Auffray et al., 2007). Macrophages are significant mediators of tissue injury and the major source of TGF-β, which are involved in inflammation and fibrosis of SSc (Toledo and Pioli 2019). Traditionally, macrophages are divided into classically activated macrophages (M1) and alternately activated macrophages (M2), which have opposite functions of pro-inflammation and anti-inflammation (Mosser and Edwards 2008). DCs have two primary subtypes—conventional dendritic cells (cDCs) and plasmacytoid dendritic cells (pDCs), both derived from the common DC precursor (Liu et al., 2009). A third subset of DCs is derived from the monocyte precursor (Guilliams et al., 2014). cDCs are a class of powerful antigen-presenting cells and consist of two principal subsets (cDC1s and cDC2s) (Guilliams et al., 2014). These cells variably express the TLRs, which are able to initiate adaptive immune responses (Boltjes and van Wijk 2014). Moreover, pDCs play a critical role in innate immunity by producing type-I IFNs (Cella et al., 1999; Siegal et al., 1999; Guiducci et al., 2010; Swiecki and Colonna 2015).

### 2.1 Monocytes

Recently, both bulk RNA-seq and scRNA-seq had confirmed the presence of a cluster of monocytes (CD16^+^ monocytes) closely related to SSc pathogenesis. Bulk RNA-seq is found as an inflammatory gene module, including *KLF10*, *JUNB*, *PLAUR*, and *JUND*, in the monocytes of the peripheral blood in SSc when compared with the healthy control (HC) (Supplementary Table S1). ScRNA-seq was further used to explore the details of monocytes, and seven subsets (PM0–PM6) were finally identified. The PM0 cluster typically expressed the abovementioned inflammatory co-expression genes and had similar transcriptomics with *IL1B* ^+^ *FCN1* ^hi^ monocytes in the lung tissue of SSc-associated interstitial lung disease (SSc-ILD) (Kobayashi et al., 2021) (Table 1). According to previous studies, *IL1B* could be a key pro-fibrotic mediator (Park et al., 2018), and *IL1B* ^+^ *FCN1* ^hi^ cells were involved in skin and lung fibrosis, indicating that the cluster might be a new therapeutic target for SSc (Aran et al., 2019). Similar results by flow cytometry showed that in addition to the activated phenotypic profile of monocytes in the peripheral blood in SSc, the number of CD16^+^ monocytes were increased and related to ILD and modified Rodnan skin score (mRSS) (Lescoat et al., 2017; Schneider et al., 2021).

**TABLE 1:** Immune cells and stroma cells in SSc by scRNA-seq.

Special subsets	Samples	Species	Diseases	Characters of special subsets	Ref.
CD16^+^ monocytes	PB	Human	SSc	Similar to IL1B^+^FCN1^hi^ monocytes in the lung of SSc-ILD	Kobayashi et al. (2021)
FCGR3A^+^ macrophages	Skin	Human	dcSSc	Derived from CCR1^+^ and MARCO^+^ macrophages both with characteristic of M1 and M2 macrophages	Xue et al. (2021)
SPP1^hi^ macrophages	Lung	Human	SSc-ILD	Encoded OPN which predicted the deterioration of lung function	Gao et al. (2020)
SPP1^hi^ and FABP^hi^ macrophages	Lung	Human	SSc-ILD	Upregulated type-I IFN signaling	Valenzi et al. (2021)
CX3CR1^+^SiglecF^+^ macrophages	Lung	Mouse	Bleomycin-induced pulmonary fibrosis	Produced nutrient factor (PDGF-aa) of fibroblasts	Aran et al. (2019)
Lyve1^hi^MHCII^lo^ macrophages	Lung, fat, heart, and dermis	Mouse	—	Restrained induced tissue fibrosis	Chakarov et al. (2019)
FCN^+^ mo-DCs	Skin	Human	dcSSc	Located in perivascular and related to severe skin fibrosis	Xue et al. (2021)
pDCs	Lung	Human	SSc-ILD	Upregulated multiple cellular stress pathways and increased the expression of type-I IFN receptor	Valenzi et al. (2021)
CXCL13^+^ T cells	Skin	Human	dcSSc	Expressed Tfh-like genes and promoted B-cell responses	Gaydosik et al. (2021)
SFRP2^hi^WIF1^+^ fibroblasts	Skin	Human	dcSSc	Precursor of myofibroblasts	Tabib et al. (2021)
Actively proliferating myofibroblasts	Lung	Human	SSc-ILD	Highly expressed collagen and other pro-fibrotic genes	Valenzi et al. (2019)
CTHRC1^+^ fibroblasts	Lung	Mouse	Bleomycin-induced pulmonary fibrosis	Expressed pathologic ECM genes and migrated into injured areas	Tsukui et al. (2020)
Activated fibroblasts	Lung	Mouse	Bleomycin-induced pulmonary fibrosis	Exhibited a myofibroblast-like gene expression signature	Peyser et al. (2019)
Endothelial cells	Lung	Human	dcSSc	Associated with ECM deposition, vascular injury, and EndMT	Apostolidis et al. (2018)

SSc, systemic sclerosis; scRNA-seq, single-cell RNA-sequencing; ILD, interstitial lung disease; PB, peripheral blood; OPN, osteopontin; Lyve1, lymphatic endothelium hyaluronan receptor-1; IFN, interferon; pDCs, plasmacytoid dendritic cells; Tfh, T follicular helper; IL, interleukin; SFRP, secreted frizzled-related protein; CTHRC, collagen triple helix repeat containing 1; ECM, extracellular matrix; EndMT, endothelial-to-mesenchymal transition.

**FIGURE 1:**
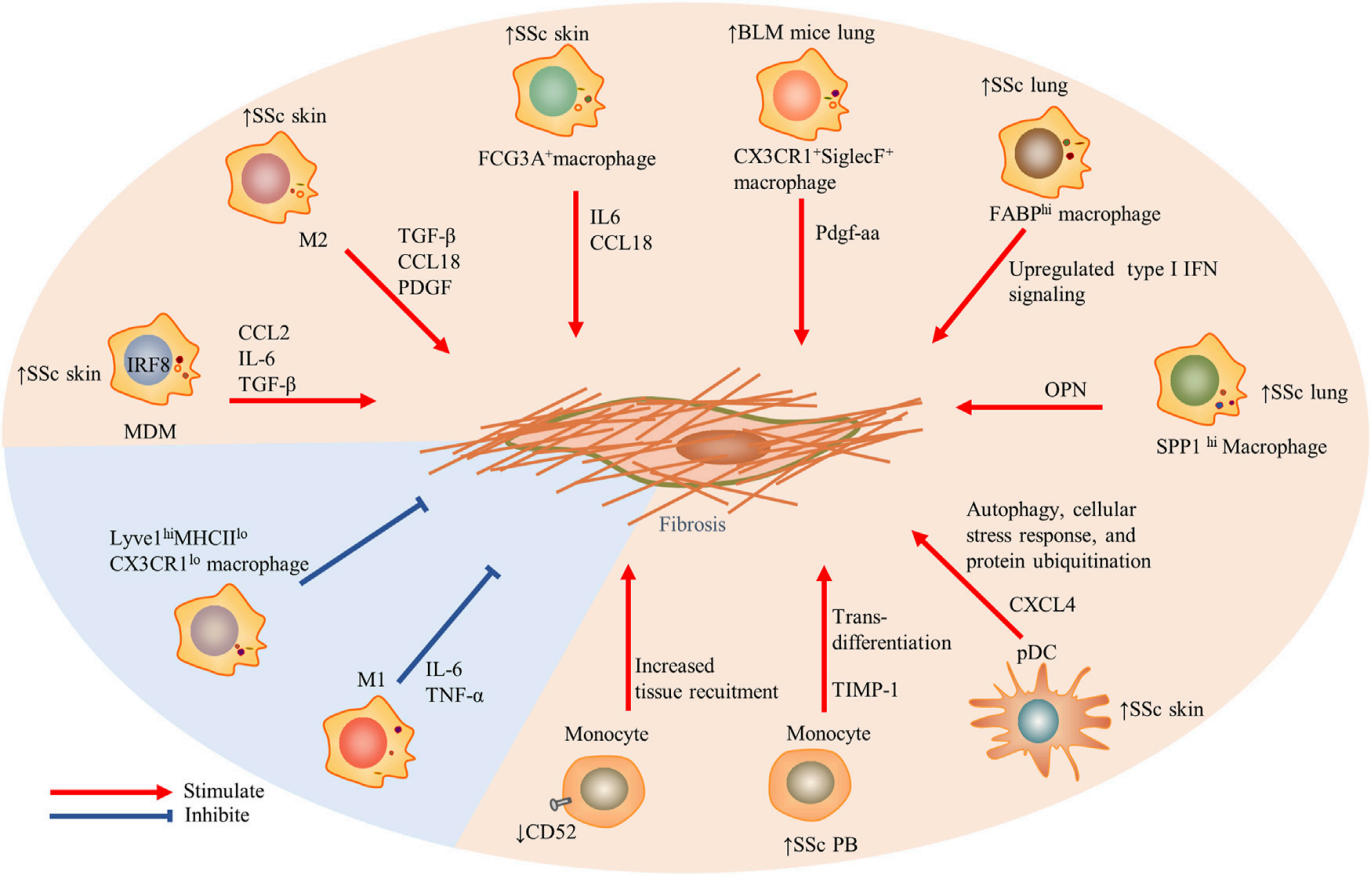
The role of innate immune cells including monocytes, macrophages, and dendritic cells in the fibrosis in SSc. Particularly, Lyve1^hi^MHCII^lo^CX3CR1^lo^ macrophages restrained induced tissue fibrosis, and traditional M1 macrophages also prevented fibrosis. Red arrows indicate stimulation; blue blunted-ends indicate inhibition. SSc, systemic sclerosis; PB, peripheral blood; IRF, interferon regulatory factor 8; MDM, monocytes-derived macrophages; CCL, CC chemokine ligand; IL, interleukin; IFN, interferon; OPN, osteopontin; TIMP-1, tissue inhibitor of metalloproteinases 1; and Lyve1, lymphatic endothelium hyaluronan receptor-1.

Recently, the whole-genome transcriptome analysis showed activated fibrotic pathway and increased fibronectin expression in circulating CD14^+^ monocytes of SSc patients. Interestingly, in a pro-fibrotic milieu, CD14^+^ monocytes were a source of ECM-producing cells, as they could differentiate into myofibroblast-like cells and produce type I collagen and *α*-SMA (Rudnik et al., 2021a) (Figure 1). One previous study described a monocyte population that secreted high levels of tissue inhibitor of metalloproteinases 1 (TIMP-1) protein, a potentially important regulator in fibrogenesis of SSc. TLR signaling had a key role in TIMP-1 secretion (Ciechomska et al., 2013). CD52 is a glycoprotein anchored to glycosylphosphatidylinositol (Xia et al., 1993; Hale 2001), and it is widely expressed on the monocytes, lymphocytes, and DCs (Ratzinger et al., 2003). Recently, a study has found that the expression of CD52 on circulating monocytes of SSc was reduced. The reduction of CD52 enhanced the adhesion between monocytes and ECs and then led to an increased recruitment of monocytes into the tissues. It is worth noting that the increased type-I IFN–related genes in SSc could downregulate the expression of CD52 in monocytes by histone deacetylase IIa (HDAC IIa). Targeting the IFN–HDAC–CD52 axis may bring a new strategy for early SSc patients (Rudnik et al., 2021b).

### 2.2 Macrophages

Robert Lafyatis et al. found five clusters of macrophages in the skin lesions of SSc patients, three of which were parallelly described in HC, including *CCR1* ^+^, *MARCO* ^+^, and *TREM2* ^+^ macrophages. Additional macrophage clusters associated with SSc were proliferating macrophages and *FCGR3A* ^+^ macrophages, and the latter were probably derived from *CCR1* ^+^ and *MARCO* ^+^ macrophages based on pseudotime analysis. *FCGR3A* ^+^ macrophages expressed M2 macrophage activation markers *CD163* and *MS4A4A* and the macrophage scavenger receptor 1 (*MSR1*) and were characterized concomitantly by the enriched pathway–like “response to lipopolysaccharide” associated with M1 polarization (Xue et al., 2020; Xue et al., 2021).

The macrophages in the SSc-ILD lung tissue were divided into seven discrete subtypes (MP1–MP7). *SPP1* expression at a higher prevalence was noted in the MP1 cluster, and the chemokine CCL18 was relatively enriched in the MP7 cluster. *SPP1* encoded osteopontin, a potential predictor for lung function deterioration. The differentially upregulated genes of the two subtypes were mainly involved in the differentiation and migration of myeloid cells and responded to pro-inflammatory signals (Gao et al., 2020). Intriguingly, subsequent studies confirmed that in addition to *SPP1* ^hi^ macrophages, three other subsets including *FABP4* ^hi^ macrophages, monocyte-derived macrophages (*FCN1* ^hi^ macrophages), and proliferating macrophages also appeared in SSc-ILD lung tissues. Compared with idiopathic pulmonary fibrosis (a chronic pulmonary fibrosis disease) and the HC, the type-I IFN signaling was upregulated in *SPP1* ^hi^ and *FABP* ^hi^ macrophages of SSc-ILD. Concretely, the interferon-induced transmembrane proteins *IF35*, *EGR1*, and *ISG15* were upregulated in the *SPP1* ^hi^ macrophages, while *TBK1*, *PTPN11*, and *CHUK* were upregulated in the *FABP4* ^hi^ macrophages (Valenzi et al., 2021).

The subpopulations of macrophages showed the greatest discrimination between mice and humans. Macrophages in the lung tissues of bleomycin-induced fibrosis mice were classified into three types by scRNA-seq: alveolar macrophages, intermediate macrophages (*CX3CR1* ^+^ *SiglecF* ^+^), and interstitial macrophages. The transitional subpopulation highly expressing MHCII molecules was located in aggregates adjacent to infiltrated *Pdgfra* ^+^ and *Pdgfrb* ^+^ fibroblasts and might be the source of Pdgf-aa (a nutrient factor of fibroblasts) in the fibrosis niche. In other words, the paracrine interactions between the macrophages and fibroblasts played a positive role in tissue fibrosis (Aran et al., 2019). Recently, *MHCII* ^hi^ *CX3CR1* ^hi^ macrophages also have been systematically studied in the lung, fat, dermis, and heart tissues of mice with a low expression of lymphatic endothelial hyaluronan receptor-1 (*Lyve1*). *Lyve1* ^lo^ *MHCII* ^hi^ *CX3CR1* ^hi^ macrophages were conserved in these tissues and located adjacent to the fibers. The other conserved macrophages were *Lyve1* ^hi^ *MHCII* ^lo^ *CX3CR1* ^lo^ macrophages with a role to restrain induced tissue fibrosis (Chakarov et al., 2019).

The traditional paradigm implies the M2 pro-fibrotic properties of monocytes/macrophages in the blood and target organs of SSc patients. M1 macrophages exerted their effects primarily at the stage of the inflammatory responses by producing IL-1β, TNF-α, and IL-6 (Gordon 2003; Mosser 2003). Infiltrated M2 macrophages in the skin and peripheral blood have been observed in SSc with the putative marker CD163 (Higashi–Kuwata et al., 2010; Bielecki et al., 2013). M2 macrophages produced pro-fibrotic mediators such as TGF-β, CCL18, and the platelet-derived growth factor to promote fibroblast activation and collagen release (Jaguin et al., 2015; Shapouri–Moghaddam et al., 2018). However, in addition to the M1 and M2 macrophages, the circulating macrophage of SSc patients also included a confluent M1/M2 phenotype which has been reported to be associated with a more severe phenotype (SSc-ILD) (Trombetta et al., 2018) and arthritis or myalgia (Mohamed et al., 2021). Monocytes in the peripheral blood in SSc might differentiate into monocytes-derived macrophages (MDMs) during exposure to the soluble factors in the plasma and secret activation markers such as CCL2, IL-6, and TGF-β. Their activation has a pro-fibrotic property (Bhandari et al., 2020), which possibly involves the key transcription factor—interferon regulatory factor 8 (IRF8), a regulator of differentiation and function of the myeloid cells. On the one hand, MDMs show an M2 phenotype after silencing IRF8; on the other hand, skin fibrosis of mice with myeloid cell–specific knockout of IRF8 increased after bleomycin treatment (Ototake et al., 2021).

### 2.3 Dendritic Cells

The results of scRNA-seq showed that there were six DC subpopulations in skin biopsies of the HC, including cDC1 (*CLEC9A* ^+^ DCs), two subsets of cDC2 (*CXorf21* ^+^ DCs and *MCOLN2* ^+^ DCs), a novel DC subtype (*LAMP3* ^+^ DCs), a cluster of proliferating DCs (*KIAA0101* ^+^ DCs), and a Langerhans cell subset (*Langerin* ^+^ DCs). *FCN1* ^+^, the monocyte-derived DC marker, was related to cDC2 in the pseudo-time distribution and existed solely in dcSSc, which was associated with severe skin fibrosis and had been mentioned in previous studies (Kobayashi et al., 2021). In addition, pDCs derived from the lymphoid progenitor cells also appeared almost exclusively in the skin in dcSSc (Xue et al., 2020; Xue et al., 2021). The upregulated pathway, including autophagy, cellular stress response, and protein ubiquitination, showed the abnormal phenotypes of activation among the pDCs of SSc-ILD (Valenzi et al., 2021).

The assay of transposase-accessible chromatin using sequencing showed that the DCs occupied the greatest epigenetic difference between SSc skin lesions and the normal skin, and the disease-related single-nucleotide polymorphisms were significantly enriched in the DCs, indicating that it may be an important epigenetic driving factor of SSc (Liu et al., 2020). The proteome-wide analysis has proved that pDCs in SSc secreted chemokine CXCL4, a predictor of mRSS (van Bon et al., 2014). pDCs expressing IFNα and CXCL4 accumulated around the vessels of the skin in SSc (van Bon et al., 2014; Ah Kioon et al., 2018). In a recent study, CXCL4, as a self-antigen, promoted the activation of type-I IFN signals by pDCs and anti-CXCL4 antibodies by B cells, which maintained the vicious circle of SSc IFN-I signature (Lande et al., 2020). Epigenetic factors such as the recent miR-126 and miR-139-5p (Chouri et al., 2021) and the previous miR-618 (Rossato et al., 2017) also contributed to the characteristics of type-I IFN in disease. Moreover, xenotransplantation of human pDCs into bleomycin-induced nonobese diabetic/severe combined immunodeficiency mice increased IFN-induced response to TLR agonist significantly and further demonstrated the key role of pDC in immune response and fibrosis degree (Ross et al., 2021).

## 3 T Lymphocytes

T-cell heterogeneity is high in terms of expression and function of T-cell receptors. Antigen-specific oligoclonal T cells are related to the breakdown in self-tolerance (Laurent et al., 2018). Specific cytokine profiles have a significant impact on the functions of fibroblasts and ECs and can promote or inhibit collagen over-synthesis and vascular diseases (Hügle et al., 2013; Chizzolini and Boin 2015). T lymphocytes are divided into T helper (Th) cells, regulatory T (Treg) cells, cytotoxic T (CTL) cells, and *γδ* T cells. All Th cells express CD4, mainly including Th1, Th2, Th17, Th9, Th22, and T follicular helper (Tfh) cells, and each subpopulation developed from naive T cells which responded to diverse microenvironmental stimuli. Treg cells primarily maintain immune tolerance. CTL cells mainly express CD8 to identify and kill abnormal target cells such as cancer cells and infected cells, but a few can also express CD4. The receptors of γδ T cell are *γ* chain and *δ* chain which permit recognition of antigens without MHC restriction. In addition, a new subset of T cells in SSc skin likely promoting B-cell responses has been fully revealed by scRNA-seq which provides a fuller framework for understanding the contribution of T lymphocytes to autoimmunity (Gaydosik et al., 2021).

### 3.1 T-Cell Subsets by scRNA-Seq

**FIGURE 2:**
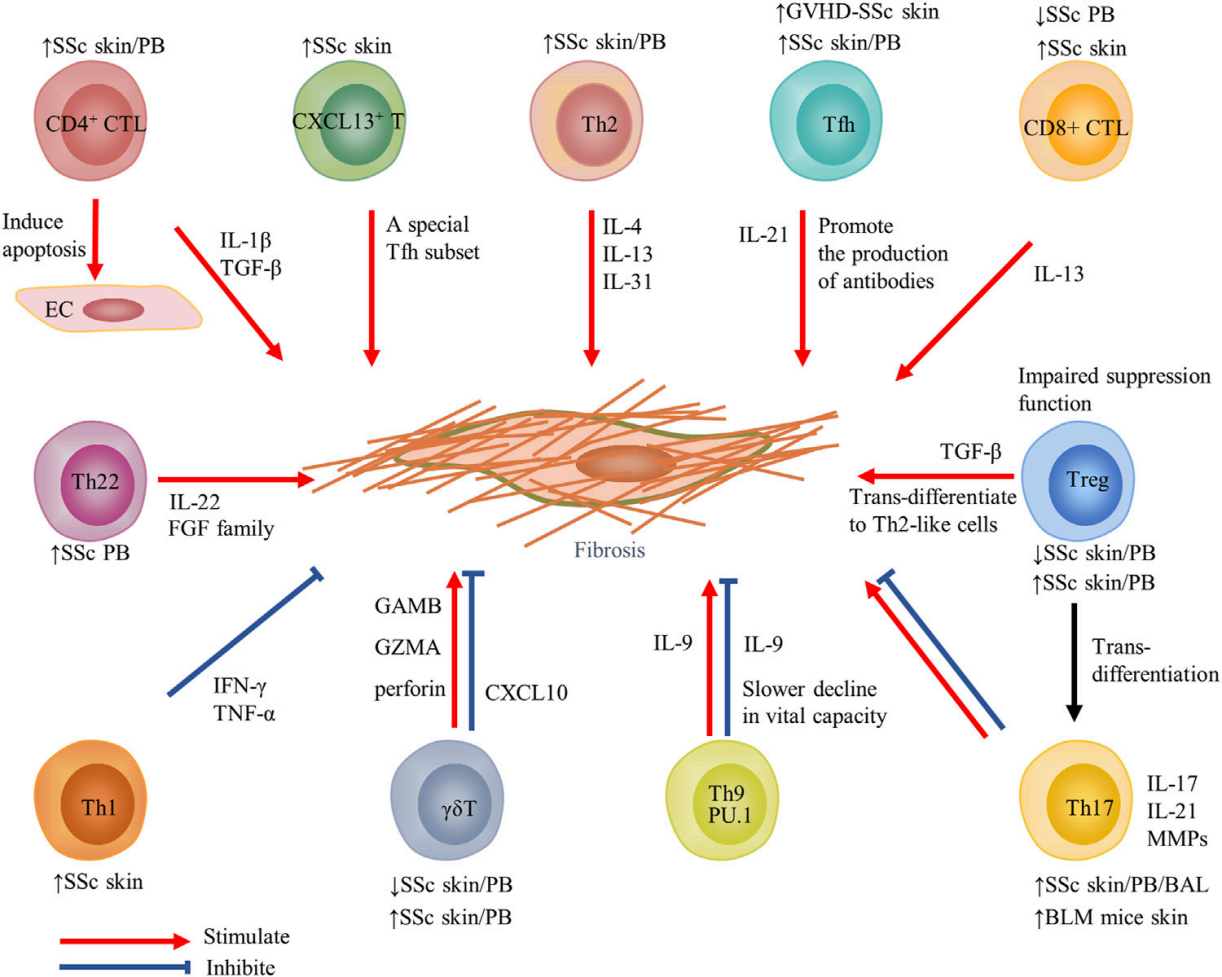
Contribution of T-cell subsets and their cytokines to fibrosis in SSc. Changes in T-cell subsets of PB or target organs in SSc and fibrosis models are displayed above or below the cell. Red arrows indicate stimulation; blue blunted-ends indicate inhibition. SSc, systemic sclerosis; PB, peripheral blood; EC, endothelial cells; IL, interleukin; Tfh, T follicular helper; GVHD, graft-versus-host disease; and MMP, matrix metalloproteinase.

Currently, only one study in 2021 analyzed the skin-resident and recirculating T-cell subsets and their heterogeneity in dcSSc by scRNA-seq. According to differences in transcriptome profiles, skin T lymphocytes were divided into nine clusters. In addition to traditional CD4^+^ T cells, CD8^+^ T cells, and Treg cells, T cell clusters expressing IL-26, proliferation genes, *TRDC*, and *CXCL13* were also identified. Each T-cell cluster had activated and special molecular pathways. Especially, the mitochondrial, ERK/MAPK signaling, and oxidative phosphorylation pathways in *CXCL13* ^+^ T cells were all upregulated. *CXCL13* ^+^ T cells expanded in the skin of dcSSc with perivascular localization and were related to ILD significantly. Moreover, the subpopulation exhibited recirculating memory migratory phenotype (*CCR7* ^+^ *SELL* ^−^), which implied a certain migration ability. *CXCL13* is a B-cell chemoattractant that helps to form tertiary lymphoid structures (TLOs) (Ansel et al., 2000). In this study, *CXCL13* ^+^ T cells were adjacent to CD20^+^ B cells and expressed Tfh-related genes with lower levels like *ICOS* and *PD1* but not *BCL6* and *CXCR5*. Therefore, it may be a special Tfh subset, and treatment for *CXCL13* ^+^ T cells may prevent disease progression (Gaydosik et al., 2021) (Figure 2).

### 3.2 Th1 and Th2 Cells

Th1 cells mainly secrete cytokines such as IFN-γ, TNF-*α*, and IL-2, while Th2 cells mainly secrete cytokines such as IL-4, IL-5, and IL-13 (Wynn 2004). At present, most researchers agree with the view of skewed Th2 pattern in SSc effector T cells (O'Reilly et al., 2012; Chizzolini and Boin 2015; Distler et al., 2019; Zhang et al., 2020; Worrell and O'Reilly 2020). Therefore, targeted delivery to restore the balance between Th1 and Th2 cytokines should be further researched for limiting fibrosis (Gourh et al., 2009). Generally, IFN-*γ* mainly inhibited collagen synthesis by reducing the activity of fibroblasts or restraining the effect of cytokines IL-4 and IL-13 (Rosenbloom et al., 1986). Compared with the HC, cytokines IL-4 and IL-13 in the serum of SSc patients were increased (Gasparini et al., 2020). IL-13 inhibited the expression of matrix metalloproteinase-1 (MMP-1) through the PKB/AKT pathway in skin fibroblasts of HC and SSc patients (Brown Lobbins et al., 2018). Latest reports have discovered the new Th2 cytokine IL-31 was elevated in plasma and target organs (skin and lung) of SSc patients (Yaseen et al., 2020). IL-31 promoted skin and lung fibrosis and enhanced Th2 immune responses, which were ameliorated by the blockade of IL-31 or anti-IL-31RA antibody (Kuzumi et al., 2021).

### 3.3 Th17 Cells

Naive T cells differentiate into Th17 cells in the presence of IL-6, IL-21, IL-23, or IL-1 (Harrington et al., 2005; Park et al., 2005; Bettelli et al., 2006), which involve inflammation and autoimmune disease by secreting IL-17, IL-22, and MMPs (Korn et al., 2009; Zambrano–Zaragoza et al., 2014). The proportion of Th17 in the blood, skin, and bronchoalveolar lavage (BAL) of patients of SSc was increased and associated with disease activity (Kubo et al., 2019; Robak et al., 2019; Fox et al., 2021). Due to the plasticity of Th17 cells, a subpopulation of Th17 cells expressed the adhesion molecule CD146 and had a stronger ability to bind and cross vascular endothelium in peripheral blood (Gabsi et al., 2019). Moreover, IFN-γ^+^IL-17^+^ Th17 cells which differentiate from Th17 in the presence of IL-12 have recently been shown to enhance the collagen secretion of fibroblasts by producing IL-21 (Annunziato et al., 2007; Xing et al., 2020). In addition, a previous study reported a significantly higher ratio of Th17/Treg cells in the peripheral blood of SSc patients than in HC, which supported the deflection towards Th17-mediated inflammatory processes (Yang et al., 2021). The conversion of Treg to Th17 cells might contribute to this bias as the CD4^+^CD25^+^FOXP3^lo^CD45RA^-^ Treg subset produced high levels of IL-17 (Liu et al., 2013).

The key cytokine IL-17 has been extensively studied controversially. IL-17 was both elevated in serum and skin during the early and active stages of SSc (Yang et al., 2014; Tezcan et al., 2021). Mechanically, IL-17 induced inflammation of ECs, and promoted fibroblast proliferation (Kurasawa et al., 2000; Xing et al., 2013). However, other sounds published their anti-fibrosis effect which reduced CTGF and type I collagen through the upregulation of miR-129-5p (Nakashima et al., 2012). More recently, a more refined organotypic model of human skin has been introduced; based on which IL-17 softened the skin by promoting inflammation and attenuating Wnt signaling (Dufour et al., 2020). It is to be noted that Th17 cells and IL-17 possessed a uniform effect in animal models which mediated fibrosis and SSc-like appearance in BLM mice and TSK-1 mice (Okamoto et al., 2012; Lei et al., 2016).

### 3.4 Th9 Cells

Th9 cells secrete IL-9 and IL-10 under the influence of pro-fibrosis factors (TGF-β, IL-4), and epithelial cytokine (thymic stromal lymphopoietin) (Veldhoen et al., 2008; Yao et al., 2013; Kaplan et al., 2015). IL-9 was highly expressed in the serum in both dcSSc and lcSSc and led to a lower degree of fibrosis and a slower decline in vital capacity at the earliest stage, which suggests IL-9 may be a protective molecule in pulmonary fibrosis (Yanaba et al., 2011). However, other studies elaborate increased IL-9 in the skin, and renal biopsy tissues were positively related to mRSS (Guggino et al., 2017). Furthermore, the Th9 transcription factor PU.1 activated the switch of pro-fibrotic fibroblast phenotype. When PU.1 was inactivated, skin and lung fibrosis in BLM-induced fibrotic mice became reduced (Wohlfahrt et al., 2019). The role of Th9 cells and IL-9 in the immune response of SSc individuals needs further research given the paucity of available results.

### 3.5 Th22 Cells

Although the existence of Th22 cells in SSc has been proved, less attention has been paid to their function. Th22 cells mainly produce IL-22 cytokine and express several members of the fibroblast growth factor (FGF) family, including FGF-1, FGF-5, and FGF-13 which play a role in tissue repair and fibrosis (Eyerich et al., 2009). It has been stressed that significantly increased circulating Th22 cells in SSc patients are related to SSc-ILD and CCR6 (a skin- and lung-homing chemokine receptor) (Truchetet et al., 2011). Furthermore, Th22 cells have been verified to participate in skin immunity because they promote keratinocyte activation induced by TNF and lead to fibroblasts obtaining a pro-inflammatory phenotype (Brembilla et al., 2016).

### 3.6 Tfh Cells

An increased frequency of Tfh cells related to disease severity in the peripheral blood of SSc patients has been observed, which promoted plasmablasts (CD19^+^CD27^+^CD38^hi^) differentiation and antibody production through the IL-21 signaling pathway (Ricard et al., 2019). Furthermore, it has been particularly emphasized that circulating Tfh cells unbalanced toward the Tfh 1 subset in SSc may alter the function of B cells through the IL-21 and IL-6 pathways. In addition, CD4^+^CXCR5^+^ Tfh cells also appeared in the skin of SSc patients (Ly et al., 2021). In the skin of graft-versus-host disease (GVHD)–SSc mice, increased ICOS^+^ Tfh-like cells promoted skin fibrosis in IL-21 and MMP-12–dependent manner (Taylor et al., 2018). Therefore, inhibition of the Tfh cell subset or their cytokines like IL-21 (ruxolitinib) may bring good news to SSc patients.

### 3.7 Treg Cells

To date, there has been little agreement on the proportions of circulating changes of SSc Tregs (Gu et al., 2008; Zhu and Paul 2008; Antiga et al., 2010; Mathian et al., 2012; Ugor et al., 2017). The possible intrinsic explanation is the decreased expression of runt-related transcription factor 1 (*Runx1*) mRNA. *Runx1* mRNA and forkhead box transcription factor (*FoxP3*) form a transcription factor complex which is essential for eliciting the suppression function of Treg cells (Kataoka et al., 2015). In SSc patients, an inflammatory environment may be harmful to the immunosuppressive functions. For instance, the frequency of activated and inactivated Treg cells was lower in SSc than in the HC, but the immunosuppressive function of SSc Tregs was restored *in vitro* (Mathian et al., 2012). In addition, co-incubation of healthy Treg cells with the plasma of SSc patients could reduce the expression of CD69 and TGF-β of Tregs by some unknown factors in the plasma, thus reducing the functional response of Treg cells to effector T cells (Radstake et al., 2009). Some studies in the target organ of SSc also had confusing results (Klein et al., 2011; Yang et al., 2014). Particularly, the circulating Treg cells could migrate to the skin tissues and become Th2-like cells due to homing molecules that secreted a large number of cytokines IL-4 and IL-13, which might have driven the differentiation of fibroblasts and lead to fibrosis in SSc patients (MacDonald et al., 2015).

### 3.8 CD8^+^ CTL Cells

Compared with HC, the proportion of circulating CD8^+^ T cells in SSc patients in the early stage was decreased and related to the prolonged course of disease (Fox et al., 2021). Although according to a 2021 study, antigen-driven expansion of CD8^+^ T cells had a high temporal persistence in the blood of SSc patients (Servaas et al., 2021). In addition, the CD8^+^ T cells lacking CD28 expression in the peripheral blood in SSc and skin lesions have been detected. It is reasonable that cytotoxic CD8^+^CD28^−^ T cells exhibited a pro-fibrotic phenotype because these cells were linked to the degree of skin fibrosis and significantly produced IL-13 (Li et al., 2017).

In recent years, genome-wide results in skin biopsies have identified CD8^+^ T cells–related genes (*CD8A*, *GraK*, *GraH*, and *GraB*) (Rice et al., 2016; Skaug et al., 2020). CD8^+^ T cells had infiltrated the skin of patients with scleroderma, particularly in the early stage, and their cytokine IL-13 promoted the activation of signal transducer and activator of transcription 6 (STAT6) signal through highly expressed receptors in the monocytes and fibroblasts (Fuschiotti et al., 2013). The growth of ECs in the skin in SSc was restricted. To some extent, this may be attributed to perforin and granzyme B expressed by CD8^+^ T cells (Kahaleh and Fan 1997; Li et al., 2017). When it comes to CD8^+^ T cells of SSc patients with pulmonary fibrosis, their proportion in the bronchoalveolar lavage fluid and lung tissue was significantly higher than for CD4^+^ T cells (Yurovsky et al., 1996; Luzina et al., 2009).

### 3.9 CD4^+^ CTL Cells

CD4^+^ CTL cells are a subset of CD4^+^ T cells with cytotoxicity, which have been widely studied in the context of chronic viral infections such as cytomegalovirus and human immunodeficiency virus. At present, significant studies gradually focused on their potential role in immune-mediated fibrosis, which promoted cytokine release, including IL-1β and TGF-β (Mattoo et al., 2016). The lymphocytes, also referred to as CD4^+^CD319^+^(*SLAMF7* ^+^) lymphocytes, were increased significantly in the peripheral blood of early dcSSc patients compared to the HC (Fox et al., 2021). CD4^+^ CTL cells labeled *GZMA* have recently been found to be the dominant infiltrating cells in the skin of patients with SSc compared with Th1, Th2, Tfh, and Tregs. Furthermore, these cells may induce apoptosis of ECs accompanied by an excessive tissue repair process leading to fibrosis and tissue dysfunction (Maehara et al., 2020), indicating the source of tissue damage mediators first discovered in skin lesions in SSc decades ago (Kahaleh and Fan, 1997).

### 3.10 γδ T Cells

The rare γδ T cells still have a place in the immune responses of the skin, intestine, and lung tissues. There were a decreased number of γδ T cells in the peripheral blood mononuclear cells (PBMCs) of scleroderma individuals, especially in patients with anti-Scl-70 antibodies and shorter disease duration (Holcombe et al., 1995). In another research, activated circulating γδ T cells of SSc patients was increased, and they could upregulate COL1A2 mRNA in co-cultured fibroblasts (Ueda-Hayakawa et al., 2013). CD27 is a co-stimulatory receptor that was very vital to the development and function of the γδ T cells. The increased pathogenicity of the CD27^+^ γδ T cells had been verified because the subset upregulated the expression of granzyme B, granzyme A, or perforin (Henriques et al., 2016). In addition, the circulating Vdelta1^+^ T cells (a γδ T subset) tended to accumulate in the skin tissues of SSc due to their expression of adhesion molecules and activation markers (Giacomelli et al., 1998). While in bleomycin-induced pulmonary fibrosis mice, γδ T cells promoted the alleviation of fibrosis by producing CXCL10 (Pociask et al., 2011). Exploring the role and potential mechanism of the minority T cell in the pathogenesis of fibrosis will be helpful for γδ T cell–based immunotherapy.

## 4 B Cells

Abundant B-cell receptors (BCRs) repertoire is generated in the bone marrow through the rearrangement of immunoglobulin gene fragments (Zhang et al., 2004). B cells are a heterogeneous population distinguished by cytokine production spectra or membrane surface molecules (Perez–Andres et al., 2010). A proliferation-inducing ligand and B-cell–activating factor (BAFF) (TNF family member) are indispensable for B-cell maturation and long-term maintenance (Mackay et al., 2003). Once naive B cells are activated by antigen or Th cells, they will differentiate into memory B cells and plasma cells which are associated with fibrosis through the production of cytokines and classical disease-specific autoantibodies in SSc, while regulatory B cells (Bregs) maintain self-tolerance primarily by cytokine IL-10 (Lee et al., 2021).

### 4.1 B-Cell Subsets by scRNA-Seq

Systematic analysis of B cells in the blood or target organs of SSc patients by scRNA-seq is a largely explored domain. We briefly describe a finding in systemic lupus erythematosus (SLE) patients. Nine clusters of B cells were identified in the PBMCs which included switched memory, naive with interferon signature (naive-Ifn), non–switched memory, switched memory-Ifn, double-negative 2 (DN2), DN2-Ifn, activated memory, naive, and plasmablast. The strong interferon signature in SLE patients may have resulted from the DN2 subset. The CD52 gene, which had a role in maintaining B-cell homeostasis, was significantly elevated in multiple B-cell clusters from SLE patients, especially in the most depleted clusters of non–switched memory B cells (Zhu et al., 2018; Bhamidipati et al., 2020).

### 4.2 Memory B Cells

Although the CD19^+^CD27^+^ memory B cells in the peripheral blood of SSc patients were decreased, the expression of their activation markers (CD80, CD95, HLA-DR) changed in the opposite direction (Forestier et al., 2018). Furthermore, recent findings have emphasized that the frequency of CD19^+^IgD^−^CD27^+^CD38^−^CD95^+^–activated switched memory (ASM) B cells in the peripheral blood of patients with dcSSc was lower than that in the HC, mostly in individuals with anti–Scl-70 antibodies or pulmonary fibrosis, implying that the ASM B cells were associated with severe SSc (Simon et al., 2021). In fact, CD21^low^ B cells were mainly composed of memory B cells such as the DN switched (CD27^−^IgD^−^) memory B cells and expressed high levels of activation markers. Beyond this, it was a possible indicator of new ulcers and visceral vascular damage (Marrapodi et al., 2020; Visentini et al., 2021).

### 4.3 Effector B Cells

Effector B cells (Beffs) participate in the immune response by secreting pro-fibrotic cytokine TGF-β (Dumoitier et al., 2017) and a variety of inflammatory markers, such as IL-4, IL-6, IL-12, TNF-α, and GM-CSF (Harris et al., 2000; Li et al., 2015; Shen and Fillatreau 2015). In the skin of SSc patients, infiltrated CD20^+^ B cells and CD138^+^ plasma cells were related to the early disease stage and disease progression (Bosello et al., 2018). The same discovery about the increases of B cells and plasma cells was verified by cutaneous transcriptome data (Whitfield et al., 2003; Streicher et al., 2018). As a classic pro-inflammatory cytokine, IL-6 promoted fibrosis by activating the downstream signaling molecule STAT3 and was also related to SSc disease severity (Sato et al., 2001). Tocilizumab (TCZ) is a humanized IL-6 receptor-α–blocking antibody (Mihara et al., 2011). Recently, the results of a phase 3 randomized controlled trial showed that TCZ stabilized the decline in forced vital capacity in SSc-ILD, whereas there was no change in mRSS (Khanna et al., 2021). The clinical end points of TCZ on skin fibrosis may be mediated only in part by the direct inhibition of fibrosis by TCZ (Khanna et al., 2018). In the bleomycin-induced scleroderma model, the BAFF inhibitor balanced the skew between Beffs and Bregs and alleviated skin and lung fibrosis (Matsushita et al., 2018). Consistent with this research, SSc patients treated with belimumab (binds with soluble BAFF with high affinity) achieved clinical improvements associated with reduced expression of B-cell activation and fibrosis-related genes in the skin (Gordon et al., 2018).

### 4.4 Regulatory B Cells

Compared with Treg cells, Bregs have relatively uniform alteration in SSc. In the peripheral blood of SSc patients, sufficient evidence indicates the decreased frequency of IL-10^+^ Bregs which is negatively related to the titers of anti-centromeric and anti-Scl-70 autoantibodies (Matsushita et al., 2016). The possible reasons for the altered quantities consists of TGF-β and IFN-γ both of which inhibited Bregs development (Iwata et al., 2011). In chronic GVHD (cGVHD) patients, Bregs enriched in the CD19^+^CD24^hi^CD38^hi^ transitional and CD19^+^IgM^+^CD27^+^ memory B cells and exhibited decreased tendencies similar to less-produced IL-10 (Khoder et al., 2014). Recently, CD24^hi^CD27^+^ Bregs were shown to be involved in the regulation of disease severity due to their reduced frequency in SSc patients with pulmonary arterial hypertension (PAH) (Ricard et al., 2021). Interestingly, T-cell Ig and mucin domain protein 1 (TIM-1), a marker of the Bregs in mice, was also identified in the human IL-10^+^ Bregs as a unique marker. TIM-1^+^ B cells in the HC inhibited the pro-inflammatory ability of CD4^+^ T cells when compared with SSc patients (Aravena et al., 2017). Animal experiments yielded similar results. For instance, early adoptive transplantation of IL-10^+^ Bregs exhibited a suppressive role in the development of sclerodermatous cGVHD (Scl-cGVHD) mice (Le Huu et al., 2013).

## 5 Fibroblasts

The activity of fibroblasts and collagen production is closely related to the pathogenesis and severity of SSc, while there are no specific markers to define fibroblasts. Recently, scRNA-seq emphasized the intrinsic transcriptome changes in fibroblasts in the skin tissues of HC and SSc patients. A total of 10 SSc fibroblast subpopulations were identified by characteristic gene expression, including *SFRP2* ^hi^ fibroblasts (*SFRP2*/*WIF* fibroblasts, *SFRP2*/*PRSS23* fibroblasts, *PCOLCE2* fibroblast), *APOE*-defined cells (*MYOC*/*FMO1*/*APOE* ^low^ fibroblasts, *CCL19*/*C7*/*APOE* ^hi^ fibroblasts), *CRABP1* fibroblasts, *COL11A1* fibroblasts, *POSTN*/*ASPN* fibroblasts, *ANGPTL7* fibroblasts, and proliferating fibroblasts. The major subpopulation was the *SFRP2* ^hi^ [secreted frizzled-related protein (*SFRP*) is related with Wnt signaling] fibroblasts located between the collagen bundles (Tabib et al., 2018). *MYOC*/*FMO1*/*APOE* ^low^ fibroblasts were distributed in the MYOC/FMO1/APOElow fibroblasts were distributed in the interstitial regions and around the blood vessels. While *CCL19*/*C7*/*APOE* ^hi^ fibroblasts showed a strong trend to SSc fibroblasts which differed from *CCL19* ^+^ fibroblasts of HC. The *CRABP1* fibroblasts, *COL11A1* fibroblasts, and *POSTN*/*ASPN* fibroblasts located around hair follicles supported previous finding about papillary dermal fibroblasts regenerating hair follicles (Driskell et al., 2013). Of note, *SFRP2*/*PRSS23* fibroblasts and *ANGPTL7* fibroblasts appeared only in SSc skin lesions. The former was associated with collagen fibril organization and ECM organization by Gene Ontology (GO) analysis, and the latter expressed *SFRP4* and represented myofibroblasts. Myofibroblasts–labeled *SFRP2*, *SFRP4*, and *FNDC1* were converted from *SFRP2* ^hi^ *WIF1* ^+^ precursor fibroblasts with the upregulation of transcriptome markers *PRSS23* and *THBS1* at the beginning. In addition, the transcription factors of fibroblasts (*RUNX1*, *FOXP1*, *IRF7*, *STAT1*, and *CREB3L1*) promoted the differentiation of fibroblasts into myofibroblasts by bioinformatics analysis (Tabib et al., 2018; Tabib et al., 2021).

The majority of the marker of pulmonary fibroblasts was not shared with dermal fibroblasts. *MFAP5* ^hi^, *SPINT2* ^hi^, few *WIF1* ^hi^ fibroblasts, and large proliferating myofibroblasts were identified between the lung tissues of the SSc-ILD and HC groups. It is noteworthy that *MFAP5* ^hi^ fibroblasts expressed higher *SFRP*, *PCOLCE2*, and *CD55* than the skin *SFRP2*/*DPP4* fibroblasts, while *WIF1* ^hi^ fibroblasts did not express *SFRP2*. In addition, myofibroblasts underwent the greatest phenotypic changes and upregulated the expression of collagen and other pro-fibrotic genes in SSc-ILD, which was pivotal to the pathologic mechanisms of fibrosis in SSc-ILD (Valenzi et al., 2019).

In the lung of bleomycin-induced fibrosis mice, researchers found *ACTA2* was not the only defined marker for activated fibroblasts. For instance, *Ltbp2*, *Col5a2*, and *Sparc* had a stronger correlation with the fibroblast activation signals. This study similarly identified the largest subcluster of fibroblasts exhibiting a myofibroblast-related gene, including muscle contraction and the development of ECM (Peyser et al., 2019). While another study has divided the fibroblasts of the lungs in bleomycin and control mice into 12 clusters, seven clusters expressed higher *COL1A1* and four clusters expressed *ACTA2*. The remaining were proliferative cells, which occupied unique anatomical locations. The study mainly found a unique type of highly expressing collagen triple helix repeat containing-1 (*CTHRC1*), which was a terminal state cluster and was detected in the multiple studies (Peyser et al., 2019; Valenzi et al., 2019; Tsukui et al., 2020). *CTHRC1* ^+^ fibroblasts mainly existed in fibrotic lungs of mice and human and expressed the highest level of type I collagen and other ECM-producing genes such as *TNC*, *POSTN*, and *COL3A1*. Purified *CTHRC1* ^+^ fibroblasts had better migration ability than other collagen-producing cells and could colonize in the lungs of mice treated with bleomycin (Tsukui et al., 2020).

## 6 Endothelial Cells

The mechanisms of EC damage and vascular disease progression in SSc are still not clear. ScRNA-seq–determined heparan sulfate proteoglycan 2 (*HSPG2*) and Apelin receptor (*APLNR*) were the two top injury markers of ECs in the skin in SSc. *HSPF2* was implicated in TGF-β signaling (Iozzo et al., 1997; Sharma et al., 1998) and SSc-associated fibrosis (Laplante et al., 2005). Due to the limitation of only one patient and one control biopsy in this study, no specific subtypes of ECs were divided. It is noticeable that enriched genes in ECs of SSc patients were related to ECM formation, vascular injury, and EndMT by the Ingenuity Pathway Analysis (IPA) and Gene Set Enrichment Analysis (GSEA) approaches (Apostolidis et al., 2018). Moreover, scRNA-seq analysis of SSc-ILD implied the active expansion of ECs in the lung tissues due to their increased vasculogenesis, prostaglandin biosynthesis, and PDGFR-signaling (Valenzi et al., 2021).

There are also other possible explanations of endothelial dysfunction. A new study found that neuronal-related characteristics such as dysregulation of the neuronal genes *ETV2* and *NRXN1* in ECs may be a culprit for dysangiogenesis in SSc (Tsou et al., 2021). In addition, sufficient evidence underlined a close liaison between vasculopathy and the metabolism of SSc dermal fibroblasts. Extracellular acidosis derived from the released lactic acid by SSc fibroblasts might lead to the impairment of peripheral capillary networks through acidic upregulated MMP-12 (an inhibitor of angiogenesis) in ECs (Andreucci et al., 2021). Intriguingly, endothelial miR-150 showed a protective effect in an animal experiment which was an independent survival predictor of PAH patients. The possible explanation implicated PTEN-like mitochondrial phosphatase (PTPMT1), which improved mitochondrial function and reduced apoptosis of ECs (Russomanno et al., 2021).

## 7 Conclusion

In SSc, the immune system and stromal cells in the blood and target organs show significant numerical or functional changes leading to the pathogenetic phenotype. ScRNA-seq provides greater insights for identifying new cell subtypes and elaborating their complex roles in disease states, and we need to further explore those subgroups that have no well-characterized functions. With the combination of conventional methods, scRNA-seq analysis, and further integrative multi-omics analysis, we could understand individual cell behavior and cellular variety, and elucidate the pathogenic mechanism of SSc diseases quickly and systematically, significantly propelling precise medical interventions in the near future.

## Glossary

AM: alveolar macrophages
anti-Scl-70: anti-topoisomerase I
APLNR: Apelin receptor
APRIL: A proliferation-inducing ligand
ASM: activated switched memory
ATAC-seq: assay of transposase accessible chromatin with sequencing
BAFF: B-cell–activating-factor
BCRs: B-cell receptors
Beffs: Effector B cells
Bregs: regulatory B cells
cDCs: conventional dendritic cells
CDP: common DC precursor
CTGF: connective tissue growth factor
CTHRC1: collagen triple helix repeat containing 1
DCs: Dendritic cells
DN: double-negative
ECM: extracellular matrix
ECs: endothelial cells
EndMT: endothelial-to-mesenchymal transition
FGF: fibroblast growth factor
FoxP3: forkhead box transcription factor
GO: Gene Ontology;
GSEA: Gene Set Enrichment Analysis
GVHD: graft-versus-host disease
HC: healthy control
HDAC IIa: histone deacetylase IIa
HSPG2: heparan sulfate proteoglycan 2;
IFN: type I interferon;
IM: interstitial macrophages
IPA: Ingenuity Pathway Analysis
IPF: idiopathic pulmonary fibrosis
IRF8: interferon regulatory factor 8
ISH: *in situ* hybridization
Lyve1: lymphatic endothelium hyaluronan receptor-1
MDMs: monocytes-derived macrophages
MMP-1: matrix metalloproteinase-1
mRSS: modified Rodnan skin score
MSR1: macrophage scavenger receptor 1
NOD/SCID: nonobese diabetic/severe combined immunodeficiency
OPN: osteopontin
PAH: pulmonary arterial hypertension
PBMCs: peripheral blood mononuclear cells
pDCs: plasmacytoid dendritic cells
PTPMT1: PTEN-like mitochondrial phosphatase
Runx1: runt-related transcription factor 1
Scl-cGVHD: sclerodermatous cGVHD
scRNA-seq: single-cell RNA sequencing
SFRP: secreted frizzled–related protein
SLE: systemic lupus erythematosus
SNPs: single-nucleotide polymorphisms
SSc: Systemic sclerosis
SSc-ILD: SSc-associated interstitial lung disease
TCRs: T-cell receptors
Tfh: T follicular helper
TGF-β: transforming growth factor-beta
Th: T helper
TIM-1: T-cell Ig and mucin domain protein 1
TLOs: tertiary lymphoid structures
TLRs: toll-like receptors
Tregs: regulatory T cells
TSLP: thymic stromal lymphopoietin
