# Supplementary material for: Contributions of Immune Cells and Stromal Cells to the Pathogenesis of Systemic Sclerosis: Recent Insights
Source: Front Pharmacol. 2022 Feb 3;13:826839. doi: 10.3389/fphar.2022.826839 (PMC8852243; doi:10.3389/fphar.2022.826839)
Supplement: Supplementary file 1 [file Table1.docx]

**Supplementary Table S1** Immune cells and stroma cells in SSc by conventional methods.

| Immune cells | Samples | Species | Diseases | Change | Characters | Ref. |
| --- | --- | --- | --- | --- | --- | --- |
| CD16^+^ monocytes | PB | human | SSc | increased | correlated with skin and pulmonary fibrosis | Lescoat et al., 2017; Schneider et al., 2021 |
| CD14^+^ monocytes | PB | human | SSc | / | had a capability to acquire a profibrotic phenotype | Rudnik et al., 2021a |
| Mixed M1 and M2 monocyte/macrophages | PB | human | dcSSc | increased | linked to pitting scars, arthritis, and myalgia | Mohamed et al., 2021 |
| MDMs | PB | human | SSc | / | induced activation of co-culture fibroblasts | Bhandari et al., 2020 |
| DCs | skin | human | SSc | increased | associated to inherited susceptibility and clinically apparent skin fibrosis | Liu et al., 2020 |
| CD146 positive Th17 cells | PB | human | SSc | increased | negatively correlated to the lung fibrosis | Gabsi et al., 2019 |
| IFN-γ^+^IL-17^+^ Th17 cells | PB/skin | human | SSc | increased | promoted fibrosis by producing IL-21 | Xing et al., 2020 |
| Th17/Treg cells ratio | PB | human | SSc | increased | / | Yang et al., 2021 |
| Th22 cells | PB | human | SSc | increased | correlated with CCR6 expression and ILD | Truchetet et al., 2011 |
| Tfh 1 cells | PB | human | SSc | increased | participated in aberrant B cell differentiation by IL-6 and IL-21 pathways | Ly et al., 2021 |
| CD4^+^CXCR5^+^ Tfh cells | skin | human | SSc | increased | / | Ly et al., 2021 |
| ICOS^+^ Tfh-like cells | skin | mouse | GVHD-SSc | increased | promoted dermal fibrosis via an IL-21 and MMP12 | Taylor et al., 2018 |
| Tregs | PB | human | SSc | decreased | / | Mathian et al., 2012 |
| Tregs | PB | human | SSc | increased | expressed CD62L and CD69 at low levels | Radstake et al., 2009 |
| Th2-like Treg cells | skin | human | SSc | increased | produced plenty of cytokine IL-4 and IL-13 | MacDonald et al., 2015 |
| CD8^+^ T cells | PB | human | dcSSc | decreased | / | Fox et al., 2021 |
| CD8^+^CD28^-^ T cells | PB/skin | human | SSc | increased | produced IL-13 and exhibited a cytolytic activity | Li et al., 2017 |
| CD8^+^ T cells | skin | human | SSc | increased | induced normal fibroblasts to produce profibrotic phenotype | Fuschiotti et al., 2013 |
| CD4^+^CD319^+^ cells | PB | human | early dcSSc | increased | / | Fox et al., 2021 |
| CD4^+^ CTL cells | skin | human | SSc | increased | induced apoptosis of endothelial cells | Maehara et al., 2020 |
| γδT cells | PB | human | scleroderma | decreased | related to anti-Scl-70 antibodies | Holcombe et al., 1995 |
| γδT cells | PB | human | dcSSc | increased | showed activated phenotype and upregulated COL1A2 mRNA expression in co-cultured fibroblasts | Ueda-Hayakawa et al., 2013 |
| Vdelta1^+^ T cells | PB/skin | human | SSc | increased | expressed activation markers and adhesion molecules | Giacomelli et al., 1998 |
| γδT cells | lung | mouse | Bleomycin-induced pulmonary fibrosis | increased | attenuated lung fibrosis by producing CXCL10 | Pociask et al., 2011 |
| CD19^+^CD27^+^ memory B cells | PB | human | SSc | decreased | activated | Forestier et al., 2018 |
| CD19^+^IgD^-^CD27^+^CD38^-^CD95^+^ ASM B cells | PB | human | dcSSc | decreased | related to anti-Scl-70 antibodies and SSc-ILD | Simon et al., 2021 |
| CD21^low^ B cells | PB | human | SSc | increased | a marker of new digital ulcers and vascular manifestations | Marrapodi et al., 2020; Visentini et al., 2021 |
| CD20^+^ B cells and CD138^+^ plasma cells | skin | human | SSc | increased | correlated with early diffuse disease and skin progression | Bosello et al., 2018 |
| IL-10^+^ Bregs | PB | human | SSc | decreased | conversely correlated with disease activity | Matsushita et al., 2016 |
| Bregs | PB | human | cGVHD | decreased | produced less IL-10 | Khoder et al., 2014 |
| CD24^hi^CD27^+^ Bregs | PB | human | SSc and SSc-PAH | decreased | took part in the regulation of cTfh and disease severity. | Ricard et al., 2021 |
| Tim-1^+^ B cells | PB | human | SSc | decreased | low suppressive ability to CD4^+^ T cell | Aravena et al., 2017 |
| Endothelial cells | skin | human | dcSSc | / | downregulated neuronal gene NRXN1 and upregulated SNAI2 and ETV2 | Tsou et al., 2021 |
| Endothelial cells | skin | human | dcSSc | / | upregulated MMP-12 and acquired mesenchymal-like features | Andreucci et al., 2021 |

SSc, systemic sclerosis; PB, peripheral blood; MDM, monocytes-derived macrophages; DCs, dendritic cells; ILD, interstitial lung disease; IL, interleukin; Tfh, T follicular helper; GVHD, graft-versus-host disease; MMP, matrix metalloproteinase; ASM, activated switched memory; cGVHD, chronic graft-versus-host disease; PAH, pulmonary arterial hypertension; Tim-1, T cell Ig and mucin domain protein 1; EndMT, Endothelial-to-mesenchymal Transition
